# Clinical significance of cerebral microbleeds in patients with germinoma who underwent long-term follow-up

**DOI:** 10.1007/s11060-024-04753-9

**Published:** 2024-08-12

**Authors:** Masayuki Kanamori, Shunji Mugikura, Osamu Iizuka, Naoko Mori, Yoshiteru Shimoda, Ichiyo Shibahara, Rei Umezawa, Keiichi Jingu, Ryuta Saito, Yukihiko Sonoda, Toshihiro Kumabe, Kyoko Suzuki, Hidenori Endo

**Affiliations:** 1 Department of Neurosurgery, Tohoku University Graduate School of Medicine, 1-1 Seiryo-machi Aoba-ku, Sendai, 980-8574 Japan; 2 Department of Diagnostic Radiology, Tohoku University Graduate School of Medicine, Sendai, Japan; 3 Department of Image Statistics, Tohoku Medical Megabank Organization, Tohoku University, Sendai, Japan; 4 Department of Behavioral and Neurology and Cognitive Neuroscience, Tohoku University Graduate School of Medicine, Sendai, Japan; 5 Department of Radiology, Akita University Graduate School of Medicine, Akita, Japan; 6 Department of Neurosurgery, Kitasato University School of Medicine, Kanagawa, Japan; 7 Department of Radiation Oncology, Tohoku University Graduate School of Medicine, Sendai, Japan; 8 Department of Neurosurgery, Nagoya University Graduate School of Medicine, Nagoya, Japan; 9 Department of Neurosurgery, Faculty of Medicine, Yamagata University, Yamagata, Japan

**Keywords:** Germinoma, Cerebral microbleeds, Brain atrophy, Intelligence, Memory, Stroke

## Abstract

**Purpose:**

This study identified the factors affecting cerebral microbleed (CMBs) development. Moreover, their effects on intelligence and memory and association with stroke in patients with germinoma who had long-term follow-up were evaluated.

**Methods:**

This study included 64 patients with germinoma who were histologically and clinically diagnosed with and treated for germinoma. These patients were evaluated cross-sectionally, with a focus on CMBs on susceptibility-weighted magnetic resonance imaging (SWI), brain atrophy assessed through volumetric analysis, and intelligence and memory.

**Results:**

The follow-up period was from 32 to 412 (median: 175.5) months. In total, 43 (67%) patients had 509 CMBs and 21 did not have CMBs. Moderate correlations were observed between the number of CMBs and time from initial treatments and recurrence was found to be a risk factor for CMB development. Increased temporal CMBs had a marginal effect on the processing speed and visual memory, whereas brain atrophy had a statistically significant effect on verbal, visual, and general memory and a marginal effect on processing speed. Before SWI acquisition and during the follow-up periods, eight strokes occurred in four patients. All of these patients had ≥ 15 CMBs on SWI before stroke onset. Meanwhile, 33 patients with < 14 CMBs or 21 patients without CMBs did not experience stroke.

**Conclusion:**

Patients with a longer time from treatment initiation had a higher number of CMBs, and recurrence was a significant risk factor for CMB development. Furthermore, brain atrophy had a stronger effect on memory than CMBs. Increased CMBs predict the stroke onset.

**Supplementary Information:**

The online version contains supplementary material available at 10.1007/s11060-024-04753-9.

## Background

Advancements in the treatments of pediatric brain tumors can lead to prolonged survival. However, patients experience late-onset adverse effects including cerebrovascular disease, neurocognitive decline, and secondary tumors [1–17]. Cerebral microbleeds (CMBs) are among the major late-onset adverse effects of the cerebrovascular system [1, 3, 4, 6].

Based on a systematic review, CMBs were found in 5% of healthy adults and were associated with hypertension and diabetes mellitus [18]. The presence of CMBs leads to the progression of mild cognitive impairment to Alzheimer’s disease [19]; attention, executive function, and fluency impairment; and cognitive dysfunction based on the Mini-Mental State Examination or the Montreal Cognitive Assessment [20, 21]. In addition, CMBs are more frequently observed in patients with stroke, particularly intracerebral hemorrhage. These lesions are a risk factor of hemorrhagic and ischemic stroke [22, 23].

Previous studies have investigated the causes and clinical manifestations of CMBs associated with pediatric brain tumors [1, 3, 5, 8, 9, 24]. Age at diagnosis, follow-up periods, histology, proton and photon-based radiation therapy, craniospinal radiation and whole-brain radiation therapy, and the administration of bevacizumab are a risk factor of CMBs [1, 6, 10, 15, 16, 24]. The association between CMBs and neurocognitive function in the survivors of pediatric brain tumors has been controversial. Some reports have revealed that the impairment of executive function [6, 8], verbal learning, and psychomotor function [8] was associated with CMBs. However, others revealed that CMBs do not affect intelligence [2]. Along with CMB development, brain atrophy was observed in the patients who underwent long-term follow-up. Although brain atrophy progresses during long-term follow-up in some of the patients with pediatric brain tumors, there were no reports of the relationship between CMBs and brain atrophy in the development of neurocognitive dysfunction.

Intracranial germ cell tumors are tumors of the central nervous system in children, adolescents, and young adults. They are histologically classified as germinomas, teratomas, yolk sac tumors, embryonal carcinomas, and choriocarcinomas [25]. Germinomas are highly sensitive to radiation and chemotherapy, and long-term tumor control has been achieved with combined low-dose radiation therapy to the whole ventricle and platinum-based chemotherapy [26–31]. The characteristics of patients with germinoma are quite different from those of patients with medulloblastoma, ependymoma, and pilocytic astrocytoma. Hence, late-onset adverse effects should be evaluated independently.

This study aimed to examine the number and distribution of CMBs in patients who received germinoma treatment at our institution. Moreover, a cross-sectional analysis was conducted to determine factors affecting the development of CMBs and their effects on intelligence and memory and stroke onset.

## Method

### Patients

This study included patients with germinoma treated at our hospital from January 1983 to July 2019. The patients were diagnosed histologically with pure germinoma and germinoma with syncytiotrophoblastic giant cells or diagnosed clinically with germinoma based on the level of tumor marker (such as serum or cerebrospinal fluid alpha-fetoprotein level of < 10 ng/mL and serum or cerebrospinal fluid human chorionic gonadotropin level of < 100 mIU/mL) and the typical findings on magnetic resonance imaging (MRI).

### Treatment and follow-up

Previous studies have reported treatment strategies for germinoma [26–29]. Radiation therapy with a dose of 40–60 Gy to local sites using a two-dimensional irradiation technique was used until 1995. Platinum-based chemotherapy alone was used from 1996 to 1997. Platinum-based chemotherapy, followed by radiation at a dose of 24 Gy to the local site with a three-dimensional irradiation technique, was utilized from 1998 to 1999. Platinum-based chemotherapy, followed by radiation to the whole ventricle at a dose of 24 Gy, was used since 2000. In addition, radiation to the whole brain or the whole craniospinal axis was performed based on the lesion extent.

After the initial treatment, the patients were followed-up for recurrence and complications. To assess the association between CMBs and stroke, we retrospectively reviewed the history of stroke from the medical records and prospectively collected the data of symptomatic stroke from study participation to December 2023.

### Data collection and acquisition and imaging data extraction

**Fig. 1:**
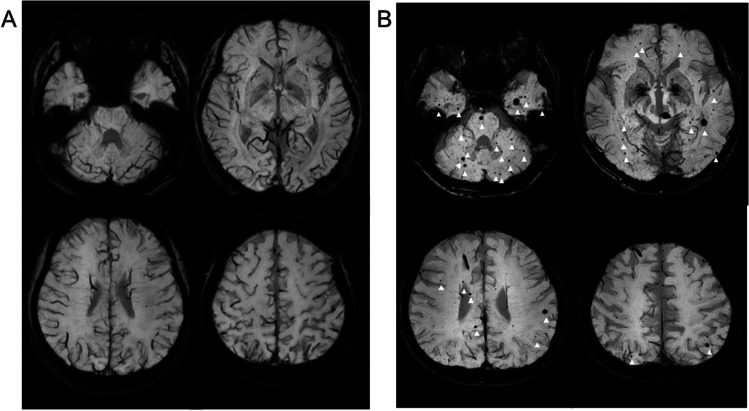
Susceptibility-weighted magnetic resonance imaging (SWI) of two patients with germinoma. **A** The representative patient without microbleeds. A 25-year-old male patient did not have any CMBs on SWI at 118 months after treatment. He was diagnosed with neurohypophyseal germinoma at the age of 15 years and was treated with cisplatin/etoposide and carboplatin/etoposide, followed by radiation therapy to the whole ventricle at a dose of 24 Gy. **B** The representative patient with microbleeds. A 40-year-old male patient had 41 CMBs in the cerebrum, basal ganglia, brain stem, and cerebellum on SWI at 317 months after treatment. He had pineal germinoma at the age of 14 years and was treated with radiation therapy to the whole brain at a dose of 30 Gy and to the primary site at a dose of 20 Gy. CMBs with more than 2 mm diameter are indicated by arrowheads

Information on the characteristics of the patients and treatment was collected from the medical records. Susceptibility-weighted magnetic resonance imaging (SWI) was performed using a 3 T MRI (Philips, Amsterdam, Nederland). The imaging conditions were as follows: 3D axial, TR: 31 ms, TE: 7.2 ms, FOV: 230 × 189 (mm), matrix: 384 × 316, FA: 17, and acquisition time: 181 s. Thirty-six minimal intensity projections with a thickness of 8 mm and − 4-mm gap reconstruction images were established. Other conditions were summarized in Supplenmental Table 1. Images were reviewed by a neurosurgeon (MK with 28 years of experience) and a neuroradiologist (SM with 30 years of experience). The two reviewers independently counted the CMBs, and the disagreements in the number of CMBs were resolved through discussion. The distribution and number of CMBs were assessed with the Microbleed Anatomical Rating Scale, which exhibits good interrater and intrarater reliabilities, according to a previous report [32]. Based on this scale, a definite CMB was defined as a lesion with a small, round, well-defined signal of 2–10 mm (Fig. 1). Linear vessel, mineralization of globus pallidus, dentate nucleus, hemorrhage within the area of infarction, and air-bone artifactwere discriminated from CMB from the finding of T2WI, FLAIR, and CT. Lesions of the cortical and subcortical white matter in the frontal, parietal, temporal, occipital, and insular lobes were classified as lobar. Otherwise, CMBs on the cingulate gyrus were separately counted to determine the effect of CMBs at these locations. Lesions of the basal ganglia, thalamus, internal and external lobes, corpus callosum, and periventricles were classified as deep. Lesions of the brainstem and cerebellum were classified as infratentorial [32]. Figure 1 shows the representative images with and without CMBs. Moreover, brain atrophy was assessed to explore its association with CMBs and neurocognitive function. Cerebral and intracranial volumes on T1-weighted images were automatically segmented using the Brainlab Elements software (Brainlab, Munich, Germany). The brain atrophy index was calculated as the cerebrum-to-intracranial volume ratio at SWI acquisition (Supplemental Fig. 1).

### Neuropsychological examination

Intelligence and memory were assessed during the study periods. Participants aged 16 years and older were assessed with the Wechsler Adult Intelligence Scale 3rd edition (WAIS-III). Participants aged younger than 16 years were evaluated with the Wechsler Intelligence Scale for Children 3rd Edition (WISC-III). The memory of participants aged 16 years and older was examined with the Wechsler Memory Scale-revised (WMS-R).

### Statistical analysis

Statistical analyses were performed with JMP Pro version 17 (Tokyo, Japan). The chi-square test was used to compare categorical variables (clinical characteristics). The Kruskal–Wallis test was used to determine the statistically significant difference in the median value between the groups. Analysis of covariance (ANCOVA) was used to compare the number of CMBs among various subgroups based on the factors that significantly differed in the univariate analysis. To evaluate the risk factors for CMB development, ANCOVA was performed with the number of CMBs as the dependent variable, radiation therapy, chemotherapy, and recurrence as independent variables, and time from the start of the initial treatment to imaging as the covariate. To analyze the association between the duration from treatment to assessment and neuropsychological test results, a linear regression analysis was performed. To elucidate the influence of CMBs on the neuropsychological examination scores, the patients were classified into the CMB-high and CMB-low groups based on the median number of overall, lobar, deep, and infratentorial CMBs as well as frontal lobe, temporal lobe, and cingulate gyrus CMBs. Similarly, they were classified into the significant brain atrophy group and nonsignificant brain atrophy groups with the median value of brain atrophy index. ANCOVA was performed with the scores for processing speed and verbal, visual, and general memory used as dependent variables, the classification with CMBs and brain atrophy as independent variables, and time from the start of the initial treatment to assessment as the covariate. *p* < 0.05 was considered to indicate statistical significance.

## Results

### Patients

In total, 146 patients were treated between 1983 and 2019. At the start of registration, 107 patients visited the outpatient department, and 64 patients agreed to participate in this study. Table 1 shows the characteristics of the patients. Male participants accounted for 81% of the study cohort. In total, 37 (50%) patients received radiation to the whole brain or the craniospinal axis. Nine (14%) patients had recurrence before registration. The follow-up period ranged from 32 to 412 (median: 175.5) months.

**Table 1:** Demographic characteristics of the patients

Age at diagnosis (years) (median)		7–37 (15)
Sex	Male:female	52:12
Location	Pineal	20 (31%)
	Neurohypophysis	16 (25%)
	Bifocal	19 (30%)
	Basal ganglia	9 (14%)
Ventricular lesion	No	48 (75%)
	Yes	16 (25%)
Chemotherapy	None	19 (30%)
	Platinum-based chemotherapy	45 (70%)
Radiation field	Local	8 (13%)
	Whole ventricle	19 (30%)
	Whole brain/craniospinal	37 (58%)
Radiation dosage to the primary site	24 Gy	30 (47%)
	40–49 Gy	14 (22%)
	50–60 Gy	20 (31%)
Recurrence	No	55 (86%)
	Yes	9 (14%)
Follow-up period (months)		32–412 (175.5)

### Features of CMB

**Fig. 2:**
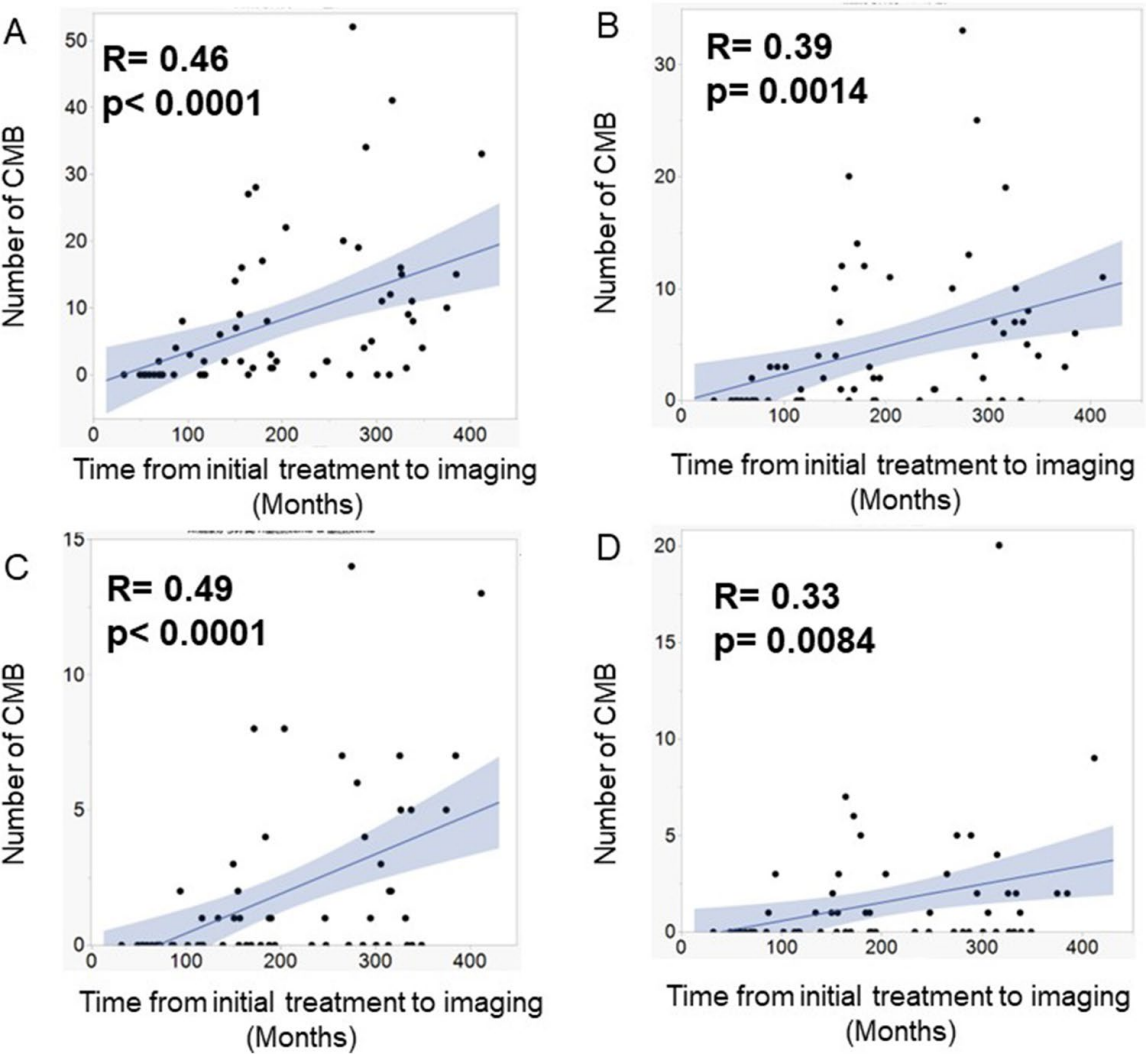
Scatter plot with linear regression and 95% confidence interval showing the association between the number of all (**A**), lobar (**B**), deep (**C**), and infratentorial (**D**) cerebral microbleeds on Susceptibility-weighted magnetic resonance imaging and the interval from the initial treatment to imaging. R = correlation coefficients. *p*-values were calculated via linear regression analysis

In total, 43 (67%) of 64 patients had CMBs. The total number of CMBs was 509 (8.0 per patient). Supplemental Table 2 shows the number of CMBs according to site. Further, 299, 94, and 116 CMBs were located in the lobar, deep, and infratentorial areas, respectively. The time from the initial treatment to MRI was moderately correlated with the total number of CMBs and the number of CMBs located in the lobar, deep, and infratentorial areas (Fig. 2). However, 21 (33%) patients did not have CMBs. The follow-up period of these patients was 55–272 (median: 72) months, and 18 patients received platinum-based chemotherapy, followed by radiation at a dose of 24 Gy using three-deminational conformal irradiation technique. Radiation field was local site in one, whole ventricle in 13 patients, whole brain in three and craniospinal axis in one. Radiation therapy was performed to the local site in one patient, the whole ventricle in 13, the whole brain in three, and the craniospinal axis in one. One patient who was not treated with chemotherapy due to cytomegalovirus infection received radiation at a dose of 24 Gy to the whole brain and at a dose of 26 Gy to the local site with three-deminational conformal radiation therapy. Contrarily, only two patients received radiation at a dose of 40–60 Gy to the local sites using a two-deminational irradiation technique.

**Fig. 3:**
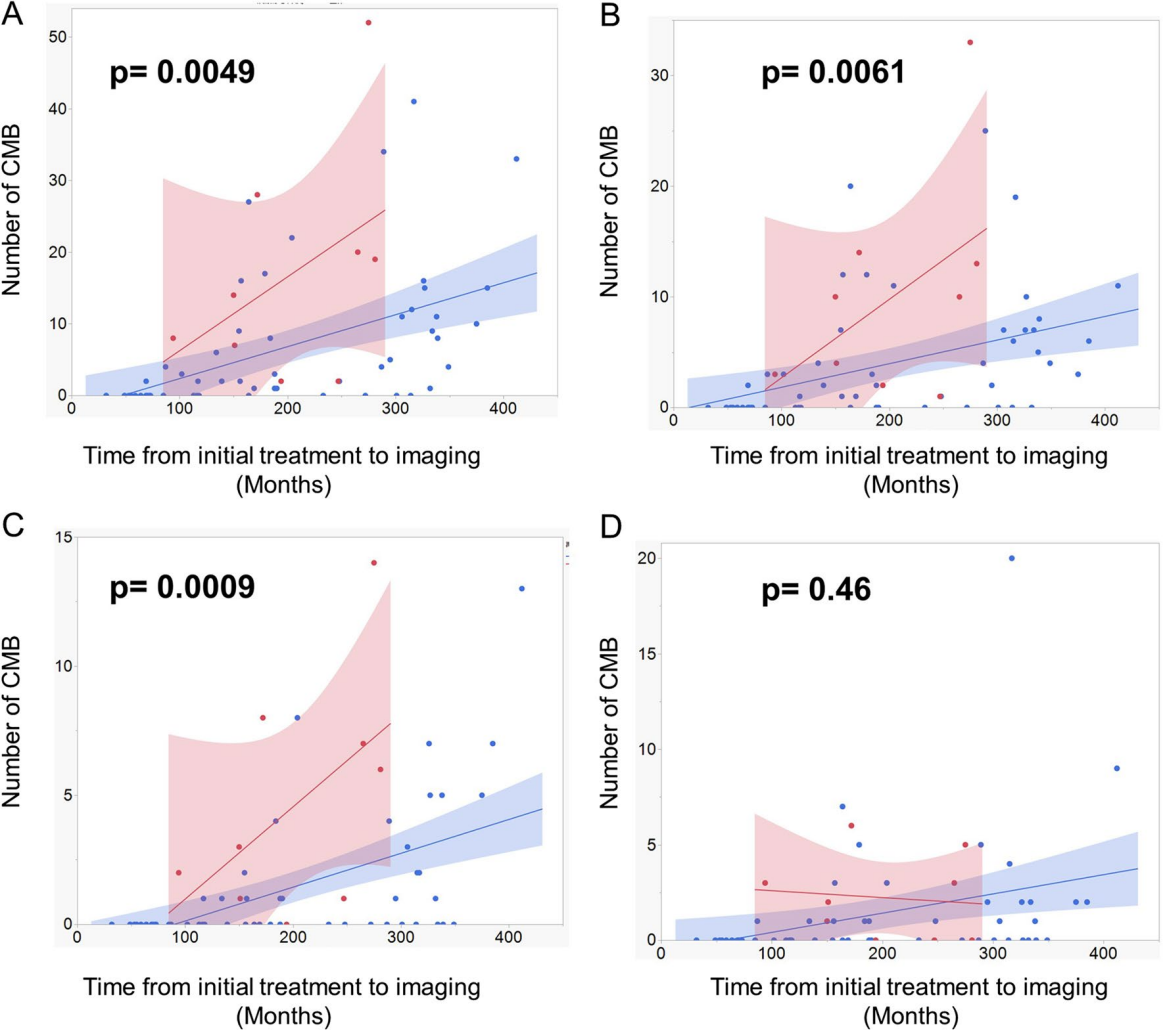
Scatter plot with linear regression and 95% confidence interval showing the association between the number of all (**A**), lobar (**B**), deep (**C**), and infratentorial (**D**) cerebral microbleeds on SWI and the interval from the initial treatment to imaging in patients with (red) and without (blue) recurrence. R = correlation coefficients. *p*-values were calculated via linear regression analysis

The risk factors of CMB development were investigated using the Kruskal–Wallis test (Supplemental Table 3). Results showed that patients who did not receive chemotherapy, those who received radiation at a dose of ≥ 40 Gy to the local site, and those who had recurrence had a higher number of CMBs. ANCOVA was performed with intervals from the initial treatment to MRI acquisition as a covariate. Among the factors with significant differences based on the Kruskal–Wallis test, patients with recurrence had a significantly higher number of overall CMBs and those in the lobar and deep areas than those without recurrence (Fig. 3). Although the results did not significantly differ, radiation at a dose of 24 Gy to the whole ventricle or the local site can reduce the development of CMBs compared with other strategies (Supplementary Fig. 2). In contrast, chemotherapy did not affect CMB development (Supplementary Fig. 3).

### Effects of CMB on intelligence and memory

Cross-sectional examinations of intelligence and memory were performed with the WAIS-III or WISC-III on 64 patients and the WMS-R on 62 patients. Supplemental Figs. 4 and 5 show the associations between scores based on these batteries and the interval from the initial treatment to evaluation. For intelligence, no correlations were found between the time from the initial treatment and verbal comprehension, perceptual reasoning, and working memory scores. However, the processing speed scores significantly decreased over time. For memory, there was no correlation between the interval from the initial treatment and attention and delayed recall scores. Nevertheless, the verbal memory, visual memory, and general memory scores significantly decreased over time.

To elucidate the association between CMB developmen and a decline in the processing speed and verbal, visual, and general memory, the patients were classified into the CMB-high and CMB-low groups with the median number of CMBs at various locations (Supplemental Table 2). The association between the interval from the initial treatment and the assessment and the score of neurocognitive accessmen was analyzed via ANCOVA. Although the temporal CMB-low group and the overall and temporal CMB-low group tended to have high processing speed and visual memoryscore (*p* < 0.10), respectively, no significant difference was observed between the other CMB-high and CMB-low groups in terms of the processing speed, as well as the general and verbal, and visual memory scores (Supplemental Figs. 6 and 7 and Supplemental Table 3).

To elucidate the influence of recurrence on neurocognitive functions, we determined whether the increase in the number of CMBs were associated with low score of neurocognitive accessments in patients with recurrence. Although all the neurocognitive functions examined tended to be lower in patients with recurrence, no statistically significant difference was observed between patients with and without recurrence in terms of the score of intelligence and memory. The representative results of processing speed and general memory are shown in Supplemental Table 4. These results indicated that the CMBs could be partially associated with the decrease in processing speed and memory and that other factors could be dominantly involved.

### Relationship of brain atrophy with CMB and neurocognitive function

**Fig. 4:**
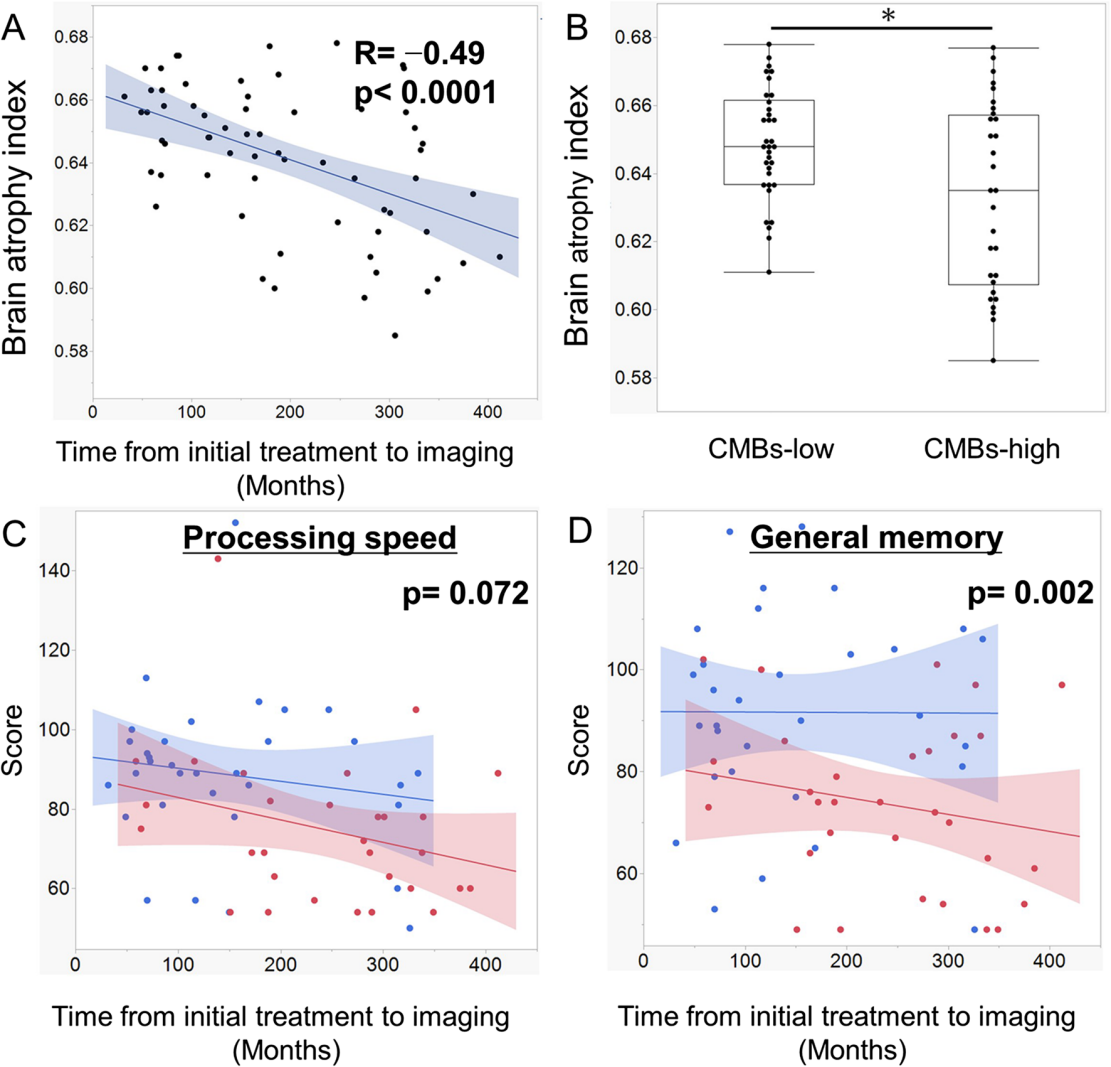
Scatter plot with linear regression and 95% confidence interval showing the association between the brain atrophy index and the interval from the initial treatment to imaging. R = correlation coefficients. *p*-values were calculated via linear regression analysis. **B** Box plot with median, quarter points, and maximum and minimum of the brain atrophy index in the lobar CMB-low and CMB-high group. * indicates *p* < 0.05. **C** and **D** Scatter plot with linear regression and 95% confidence interval demonstrating the association between th processing speed (**C**) and general memory score (**D**) and the interval from the initial treatment to imaging in patients with significant brain atrophy group (red) and nonsignificant brain atrophy groups (blue). *p*-value was calculated via analysis of covariance

We further analyzed the association between lobar CMBs and brain atrophy. The brain atrophy index ranged from 0.595 to 0.678 (median: 0.646). It exhibited a moderately negative correlation with time from the initial treatment to imaging (Fig. 4). Furthermore, the brain atrophy index was significantly lower in the lobar CMB-high group than in the lobar CMB-low group (median: 0.635 vs 0.648; *p* = 0.037; Kruskal–Wallis test) (Fig. 4). All of the 10 patients with brain atrophy index ≤ 0.610 were classified into the lobar CMB-high group (Fig. 4). These results indicated that brain atrophy progressed over time and that it was more severe in the lobar CMB-high group.

We classified the patients by the median value of the brain atrophy index and examined the effect on the processing speed and memory through ANCOVA (Supplemental Table 4). The severe brain atrophy group had significantly low verbal, visual, and general memory scores and tend to have low processing speed score (Fig. 4).

### Association between CMB and stroke

In total, 4 of 64 patients developed eight strokes (Supplemental Table 5). None of the patients had familial history related to stroke or smoking. The comorbidities were hyperlipidemia (*n* = 2), hypertension (*n* = 1) and panhypopituitarism (*n* = 1). The interval between the date of the initial treatment and the first stroke ranged from 98 to 408 months. All patients received radiation therapy at a dose of 50 Gy to the local site with two-dimensional planning. Stroke occurred three times in one patient and twice in two. Four patients presented with ischemic and four with hemorrhagic strokes.

The association between the SWI findings and the onset of stroke was examined. Before SWI acquisition, two patients had a history of stroke (cases 1 and 2 in Supplemental Table 5). Case 1 had the lesion at neurohypophysis, and received radiation therapy and chemotherapy at 15 years-old. He suffered thalamic hemorrhage and pontine hemorrhage 98 and 191 months after the initial treatment, respectively. SWI acquired 289 months after the treatment showed 34 CMBs. Case 2 had the lesion at the pineal regio and received radiation therapy at 22 years- old. He developed lacunar infarction 337 and 376 months after treatment, and hemorrhage at caudate nucleus 402 months after initial treatments. SWI acquired 412 months after the treatment showed 33 CMBs. To evaluate the significance of CMBs for future stroke prediction, we reviewed their T2*-weighted gradient-echo sequence (T2*GRE) images. As a result, 16 and 17 CMBs were detected in the T2*GRE images 32 months before the onset of the second stroke in case 1 and at the onset of the first stroke in case 2, respectively. After SWI acquisition,all but one, who did not visit for follow-up after imaging, patients were followed-up from 5 to 96 (median: 63) months. During this period, two patients who had 15 and 20 CMBs developed stroke (cases 3 and 4 in Supplemental Table 5). Of note, 51 patients with < 14 CMBs, including 33 patients with < 14 CMBs or 18 patients without CMBs, did not have stroke before SWI acquisition and the follow-up periods. These results indicated that ≥ 15 CMBs could predict future stroke onset.

## Discussion

Germinoma has various characteristic features of age at onset and tumor location. Moreover, they can be managed with treatment strategies including low-dose and large-field radiation therapy and platinum-based chemotherapy, and have an excellent survival time. A previous study evaluated the T2*GRE findings of 34 patients commonly treated with radiation therapy at a dose of 50 Gy to the primary site with two-dimensional planning [24]. In that studyresearch, the median follow-up period after treatment was 18.5 years. The frequency of CMBs was high at 94.1%, and the incidence of CMBs in the whole-brain irradiation group was significantly high. In addition, the incidence of CMBs in the high-dose area was higher. Our previous study had two drawbacks. One was thatthe association between the intervals from the initial treatment to imaging and neuropsychological function was not examined. The other was that our previous study did not include patients managed with a new treatment strategy, particularly radiation at a dose of 24 Gy to the whole ventricle combined with platinum-based chemotherapy [26, 33]. Thus, we analyzed the association between CMBs and neurocognitive function, as will be discussed later, as well as the change in CMB development in patients treated with the current strategies. As regards the latter subject, only one study has reported the development of late-onset vascular adverse events caused by these treatments. In particular, CMBs were found in four of five patients [33]. In this study, CMB was not observed in 33% of the patients, and 85.7% of the patients were treated with low-dose ventricular or whole-brain irradiation with platinum-based chemotherapy. Further, based on the number of CMB adjusted by the intervals from the initial treatment with ANCOVA, this treatment strategy may reduce the development of CMBs.

Although the effects on the neurocognitive function of recurrence is not conclusive, recurrence was a risk factor for the development of CMBs based on the Kruskal–Wallis test and ANCOVA. Eight of nine patients received reirradiation at the time of recurrence, and this salvage treatment could be attributed to the development of CMBs. We previously reported that patients who initially received high-dose irradiation or repeated salvage radiation presented with a decline in intelligence and social life. However, salvage radiation to the craniospinal axis at a dose of 24 Gy can preserve them in patients who received radiation to the whole ventricle at a dose of 24 Gy plus chemotherapy [27]. Two patients treated with the former strategy had > 20 CMBs. Meanwhile, three of five patients treated with the latter strategy had < 10 CMBs. Based on these results, recurrence itself was a risk factor for CMB development. Nevertheless, salvage treatment with the latter strategy can prevent the development of CMBs by reducing the total radiation dosage.

Some reports have evaluated the neurocognitive function of patients after treatment with reduced-dose radiation therapy to the extended field including the whole craniospinal axis, whole brain, or whole ventricle for germinoma. Cheng et al. reported the longitudinal changes in intelligence in eight patients with germinoma treated with radiation at a dose of 23.4 Gy to the extended field or at a dose of 40 Gy to the local site [34]. This study did not show that radiation to the extended field at a dose of 24 Gy had effects on intelligence due to the small number of patients (*n* = 4 in each group) and the short follow-up period with a mean interval of 38.4 months. Lee et al. reported that patients who received radiation at a dose of 30 Gy to the extended field presented with a decline in neurocognitive function [35]. Marbott et al. evaluated the longitudinal neurocognitive function of 35 patients with a mean follow-up period of 3.3 years [36]. In their study, working memory, processing speed, and visual memory declined over time. Moreover, female sex, radiation to the whole brain, radiation dose of > 30 Gy, younger age at onset, and hydrocephalus were risk factors of late-onset adverse effects on neurocognitive function [36]. In the current study, patients with long-term follow-up had lower processing speed, verbal memory, visual memory, and general memory scores. This result is consistent with those of previous studies. In particular, the decline in processing speed was significant. Processing speed is a measure that captures the ability to process information rapidly [37]. Along with working memory, which refers to the ability to retain information for short periods of time, it is a function that is more likely to be impaired in the long-term survivors of pediatric cancer. These impairments can reduce learning social development, academic performance, and future vocational attainment, thereby directly affecting the patient’s social functioning [38–40]. In an analysis of long-term survivors after treatment for pediatric brain tumors, multiple factors have been found to affect processing speed. These include age at onset, radiation therapy, radiation dosage to the supratentorial lesion, cochleae, optic nerve, cerebellum, vermis and pons, resection, and chemotherapy [13, 41, 42]. Small vessel lesions such as CMBs and lacunar infarcts, which involve white matter damage, and decreased regional cerebral blood flow [11, 12, 38] are also the underlying pathologies of processing speed decline. Roddy et al. demonstrated the assocoation between neurocognitive dysfunction and the location of CMBs in patients with pediatric brain tumor. The presence of CMBs in the frontal and temporal lobes were associated with the impairment of executive function and working memory and verbal learning, respectively [6]. In this study, increased temporal CMBs exerted marginal effects on process speed and memory scores, but the effect of CMBs on neurocognitive function seemed modest. As the patients’ background and the assessment tool used in this study were quite different from those in our study, further examination is warranted to elucidate the relationship between neurocognitive dysfunction and CMB location.

Meanwhile, we examined the effects of brain atrophy on neurocognitive function. Based on the cross-sectional observation, our study first demonstrated the association between CMBs and brain atrophy and their relative effects on neurocognitive functions. Both brain atrophy and CMBs progressed over time. Brain atrophy dominantly impacted the memory score and had a marginal effect on processing speed as well as temporal CMBs. Currently, studies investigating the influence of brain atrophy on neurocognitive function and CMBs are scarce. Kline et al. demonstrated that the presence of CMBs and the volume of white matter lesions (WML) after radiation therapy to brain tumors were independently associated with a low executive function score [8]. In this study, the nature of WML was quite different from brain atrophy. WML was defined as an abnormally high signal on T2WI, with findings in 95% of cases at an average of 4.3 years after radiation therapy, whereas brain atrophy was defined as the decreased volume of the cerebrum with a median follow-up duration of 22.1 years in this study.

Stroke is an important late-onset adverse effect after the treatment of pediatric brain tumors. A longitudinal observation with a median of 23.0 years from the diagnosis of 1876 pediatric brain tumors revealed that the hazard ratio for stroke compared with their siblings was 14.9 [43]. Balve et al. performed a systematic review of stroke after radiation therapy to pediatric brain tumors [44]. Most patients with stroke had suprasellar tumors including optic pathway glioma and craniopharyngioma.The risk factors of stroke were dose to the optic chiasm or circle of Willis and neurofibromatosis type 1. To the best of our knowledge, this is the first report on the association between CMBs and stroke in patients with germinoma who had long-term follow-up. All patients received radiation to the primary site at a dose of 50 Gy with two-dimensional radiation planning and developed stroke at the basal ganglia or thalamus. In total, 4 (31%) of 13 patients with ≥ 15 CMBs had stroke before or after the acquisition of SWI. Although the threshold of the number of CMBs remains unclear, the number of CMBs can predict the development of stroke.

The current study had several limitations. First, it is cross-sectional study in the patients managed with different treatments and lacks the longitudinal changes. However, we can analyze data on the longitudinal changes in CMBs in some cases who participated in our previous cross-sectional study of CMBs [24]. That cross-sectional study on germinoma conducted from August 2006 to December 2013. The patients received 50 Gy of radiation therapy to the local site with two-dimensional irradiation technique and were evaluated using T2*GRE images. Of the patients, 19 participated in the second study. The median interval between the first and second imaging ranged from 48 to 139 (median: 107) months. Although it is difficult to compare the number of CBMs due to different detection profiles between T2*GRE and SWI [45], the number of CMBs increased in 15 of 19 patients, and such an increase ranged from 1 to 29 with a median of 5. In the future, the effect of reduced-dose radiation therapy on CMB development can be elucidated by comparing the findings of SWI and neurocognitive data in this study. Second, this study did not investigate the association between radiation dosage and the development of CMBs based on the dose distribution on three-dimensional conformal radiation therapy or intensity-modulated radiation therapy. These analyses should validate the definitive causality between radiation dosage and the development of CMBs Third, this study did not demonstrate the direct causal relationship between neurocognitive function impairment and CMBs or brain atrophy. Considering the significant long intervals and differences in the treatment protocols, there might be other confounding factors causing neurocognitive function impairment that have not yet been discovered.

## Conclusion

The number of CMBs increased over time based on the long-term follow-up. Recurrence was a significant risk factor of CMB development, and reduced-dose radiation therapy to the whole ventricle could reduce its development. Processing speed and verbal, visual and general memory were impaired in patients with long-term follow-up. Brain atrophy could be associataed with the decline in memory than CMBs. The increased number of CMBs could predict stroke onset.

## Supplementary Information

Below is the link to the electronic supplementary material.

Supplementary file1 (XLSX 11 kb)

Supplementary file2 (XLSX 10 kb)

Supplementary file3 (XLSX 21 kb)

Supplementary file4 (XLSX 12 kb)

Supplementary file5 (XLSX 12 kb)

Supplementary file6 (DOCX 871 kb)

## Data Availability

No datasets were generated or analysed during the current study.
