# Supplementary material for: Clinical significance of cerebral microbleeds in patients with germinoma who underwent long-term follow-up
Source: J Neurooncol. 2024 Aug 12;170(1):173–84. doi: 10.1007/s11060-024-04753-9 (PMC11447146; doi:10.1007/s11060-024-04753-9)
Supplement: Supplementary file 6 — Supplementary file6 (DOCX 871 kb) [file 11060_2024_4753_MOESM6_ESM.docx]

**Supplementary Material**

**­­­­­Clinical significance of cerebral microbleeds in patients with germinoma who underwent long-term follow-up**

**Journal of Neuro-Oncology**

Masayuki Kanamori, MD, PhD^1^, Shunji Mugikura, MD, PhD^2,3^, Osamu Iizuka, MD, PhD^4^, Naoko Mori, MD, PhD^5^, Yoshiteru Shimoda, MD, PhD^1^, Ichiyo Shibahara, MD, PhD^6^, Rei Umezawa, MD, PhD^7^, Keiichi Jingu, MD, PhD^7^, Ryuta Saito, MD, PhD^8^, Yukihiko Sonoda, MD, PhD^9^, Toshihiro Kumabe, MD, PhD^6^, Kyoko Suzuki, MD, PhD^4^, Hidenori Endo, MD, PhD^1^

Affiliations:

^1^Department of Neurosurgery, Tohoku University Graduate School of Medicine, Sendai, Japan

^2^Department of Diagnostic Radiology, Tohoku University Graduate School of Medicine, Sendai, Japan

^3^Department of Image Statistics, Tohoku Medical Megabank Organization, Tohoku University, Sendai, Japan

^4^Department of Behavioral and Neurology and Cognitive Neuroscience, Tohoku University Graduate School of Medicine, Sendai, Japan

^5^Department of Radiology, Akita University Graduate School of Medicine, Akita, Japan

^6^Department of Neurosurgery, Kitasato University School of Medicine, Kanagawa, Japan

^7^Department of Radiation Oncology, Tohoku University Graduate School of Medicine, Sendai, Japan

^8^Department of Neurosurgery, Nagoya University Graduate School of Medicine, Nagoya, Japan

^9^Department of Neurosurgery, Yamagata University Faculty of Medicine, Yamagata, Japan

Corresponding author: [mkanamori@med.tohoku.ac.jp](mailto:mkanamori@med.tohoku.ac.jp)


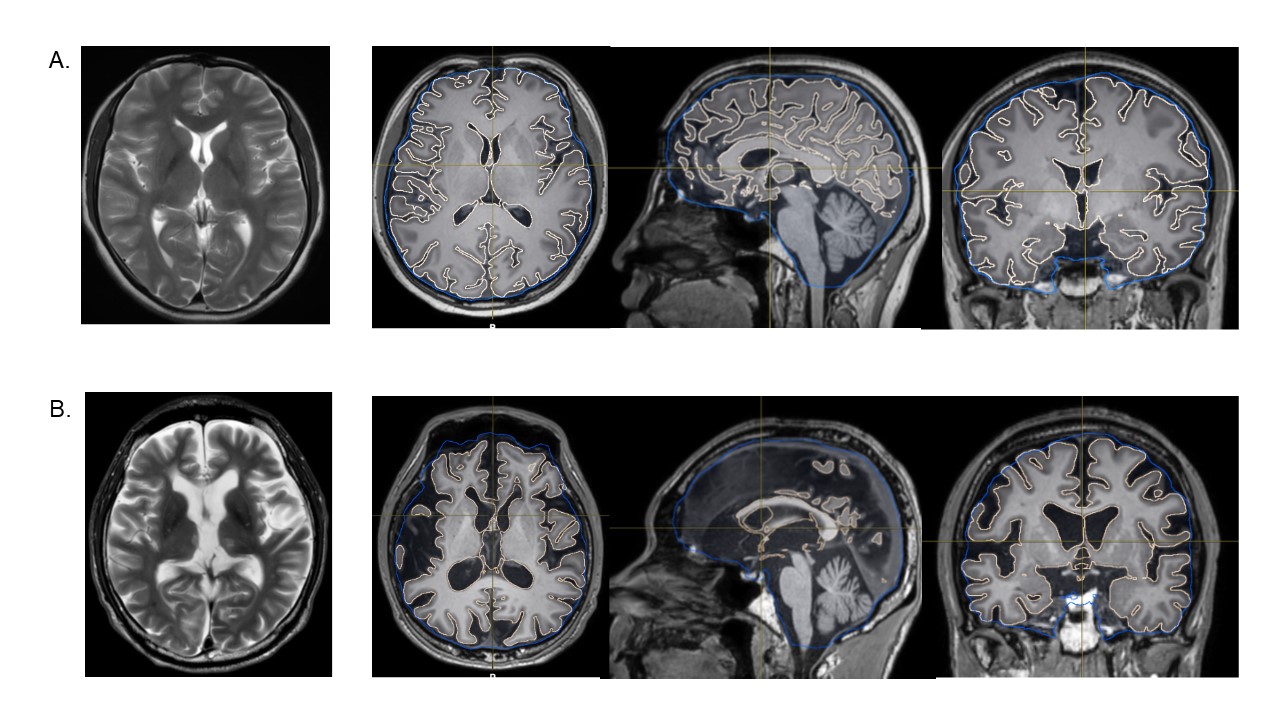


Supplemental Fig. 1. Representative cases for the assessment of brain atrophy. T2-weighted magnetic resonance images (T2WI) (left panels) and T1-weighted images with automatically segmented cerebrum (pale orange) and intracranial space (blue) (right panels). The brain atrophy index was calculated as the cerebrum-to-intracranial volume ratio. A. 32-year-old woman with neurohypophysis germinoma 21 years after treatment. The brain atrophy index was 0.678. B. A 48-year-old man with pineal germinoma 36 years after treatment. The brain atrophy index was 0.585.


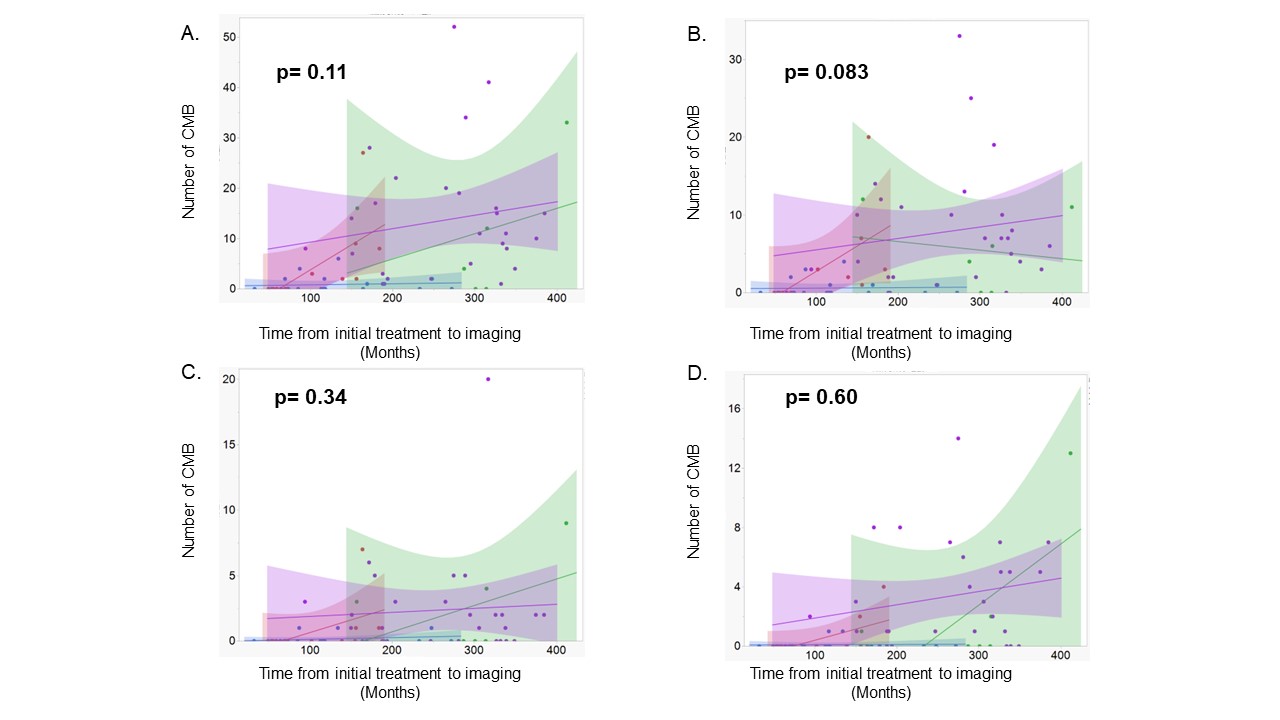
Supplemental Figure 2. Scatter plot with linear regression and 95% confidence interval showing the association between the number of all (A), lobar (B), deep (C), and infratentorial (D) cerebral microbleeds on susceptibility-weighted magnetic resonance imaging and the interval from the initial treatment to imaging in patients treated with reduced-dose radiation to the whole ventricle or primary site (blue), reduced-dose radiation to the whole craniospinal axis or the whole brain (orange), high-dose radiation to the primary site (green), high-dose radiation to the primary site and radiation to the whole brain or the craniospinal axis. p-value was calculated via analysis of covariance.


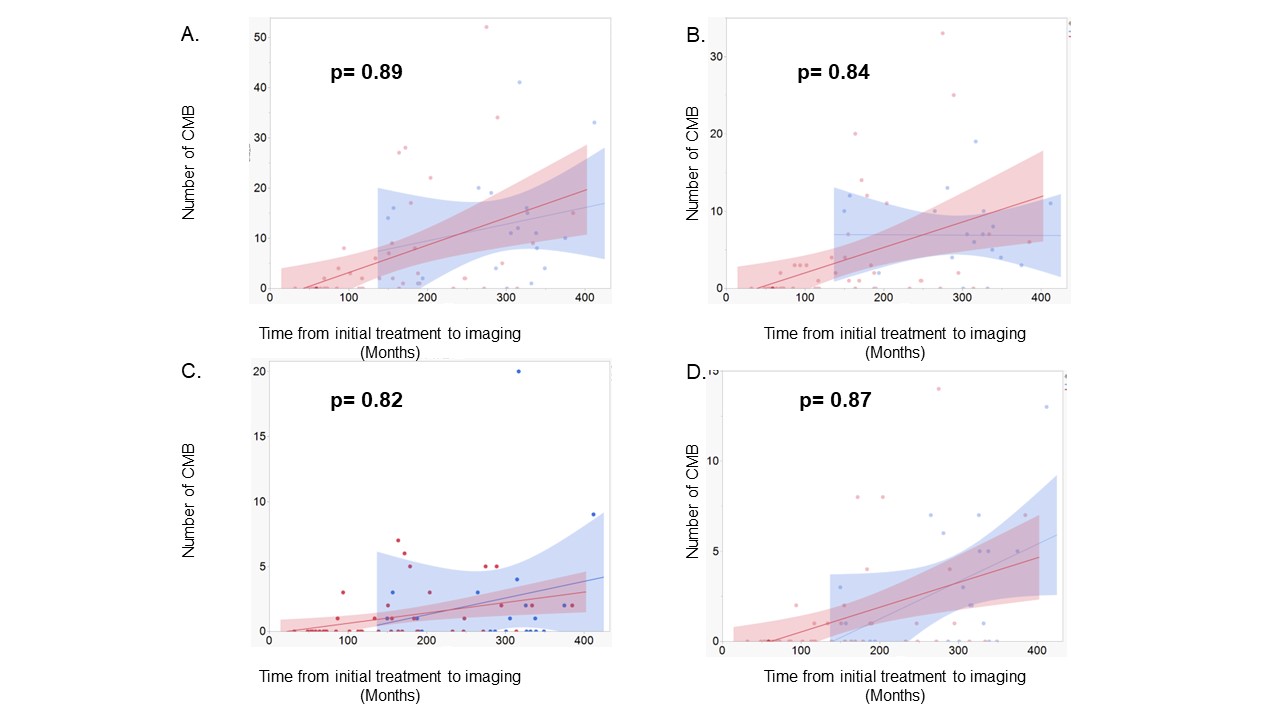


Supplemental Figure 3. Scatter plot with linear regression and 95% confidence interval showing the association between the number of all (A), lobar (B), deep (C), and infratentorial (D) cerebral microbleeds on susceptibility-weighted magnetic resonance imaging and the interval from the initial treatment to imaging in patients treated with (red) and without (blue) chemotherapy. p-value was calculated via analysis of covariance.


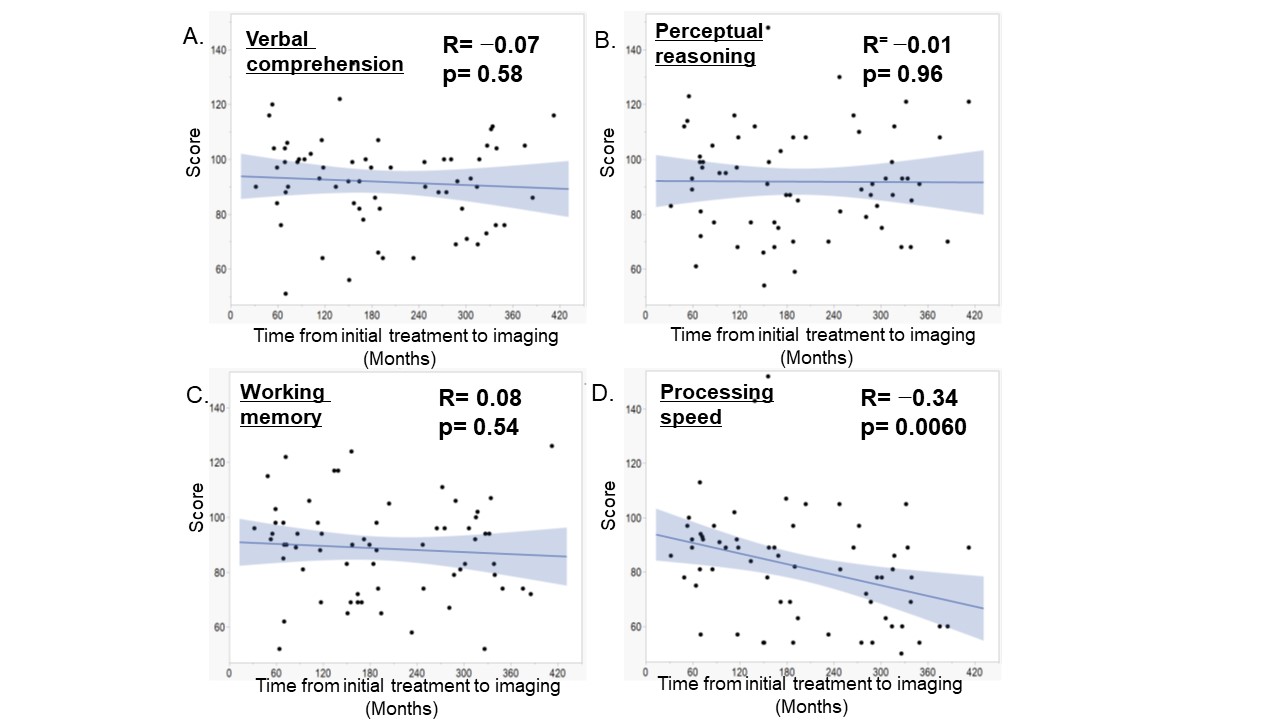
Supplemental Figure 4. Scatter plot with linear regression and 95% confidence interval showing the association between the score for verbal comprehension (A), perceptual reasoning (B), working memory (C), and process speed (D) and the interval from the initial treatment to imaging in patients with germinoma. p -value was calculated via linear regression analysis.


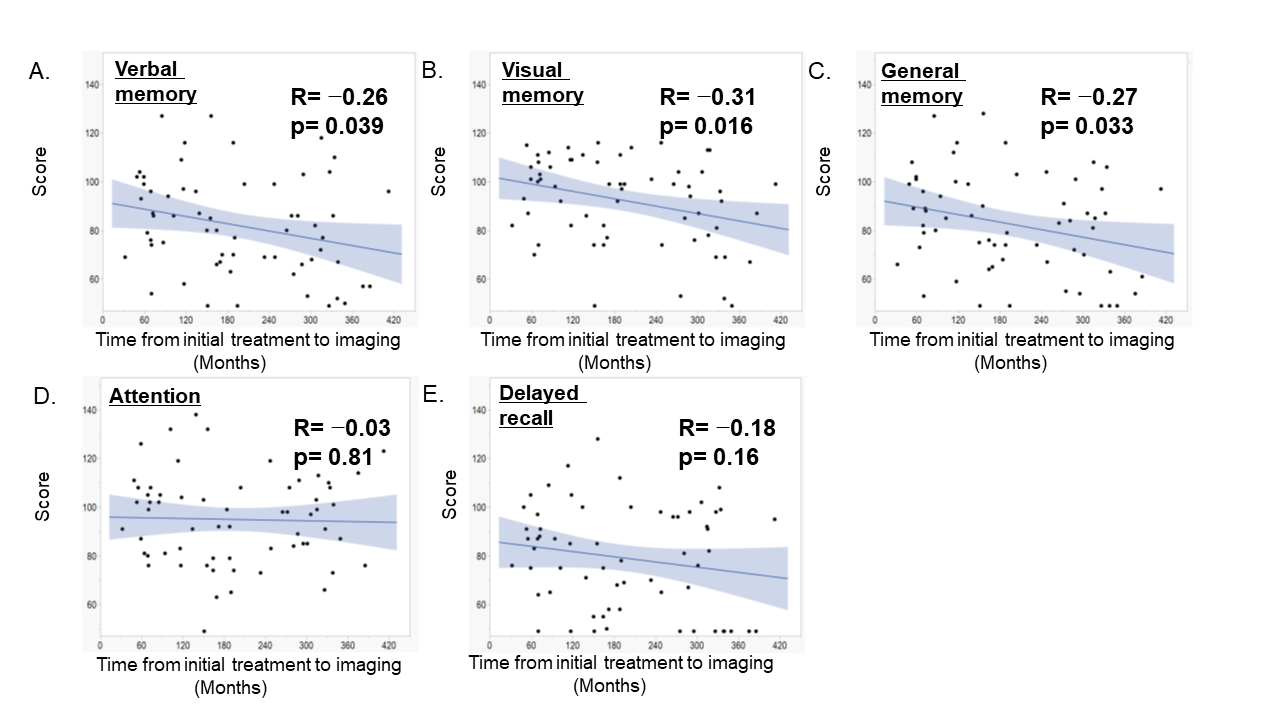
Supplemental Figure 5. Scatter plot with linear regression and 95% confidence interval showing the association between the score for verbal memory (A), visual memory (B), general memory (C), attention (D), and delayed recall (E) and the interval from the initial treatment to imaging in patients with germinoma. P-value was calculated via linear regression analysis.


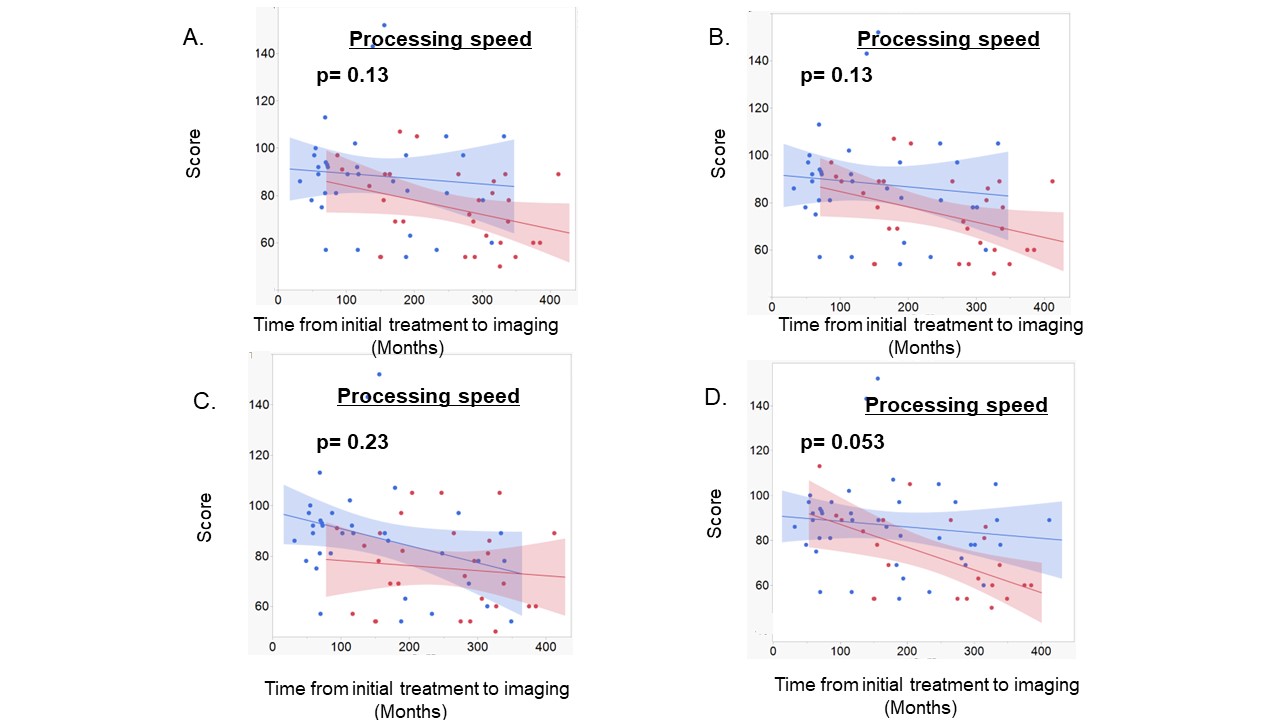


Supplemental Figure 6. Scatter plot with linear regression and 95% confidence interval showing the association between the processing speed and the interval from the initial treatment to assessment in patients with greater than (red) or less than or equal (blue) to the median number of all (a), lobar (b), deep (c), and temporal (d) cerebral microbleeds. p-value was calculated via analysis of covariance.


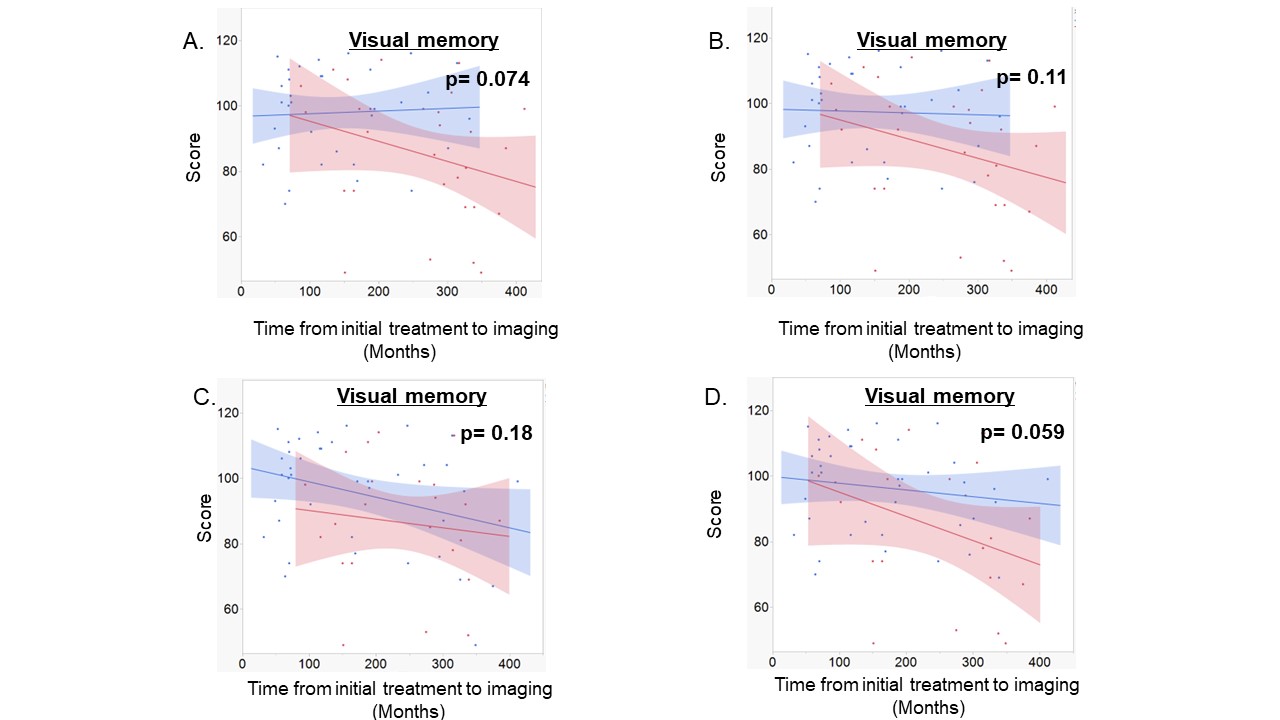
Supplemental Figure 7. Scatter plot with linear regression and 95% confidence interval showing the association between the score for visual memory and the interval from the initial treatment to assessment in patients with greater than (red) or less than or equal (blue) to the median number of all (A), lobar (B), frontal (C), and temporal (D) cerebral microbleeds. P-value was calculated via analysis of covariance.
